# Serologic Evidence of Human Exposure to Ehrlichiosis Agents in Japan

**DOI:** 10.3201/eid2811.212566

**Published:** 2022-11

**Authors:** Hongru Su, Kenji Kubo, Shigetoshi Sakabe, Shinsuke Mizuno, Nobuhiro Komiya, Shigehiro Akachi, Hiromi Fujita, Kozue Sato, Hiroki Kawabata, Hiromi Nagaoka, Shuji Ando, Norio Ohashi

**Affiliations:** University of Shizuoka, Shizuoka, Japan (H. Su, N. Ohashi);; Japanese Red Cross Wakayama Medical Center, Wakayama, Japan (K. Kubo, S. Mizuno, N. Komiya);; Ise Red Cross Hospital, Ise, Japan (S. Sakabe);; Mie Prefecture Health and Environment Research Institute, Yokkaichi, Japan (S. Akachi);; Mahara Institute of Medical Acarology, Anan, Japan (H. Fujita);; National Institute of Infectious Diseases, Tokyo, Japan (K. Sato, H. Kawabata, S. Ando);; Shizuoka Institute of Environment and Hygiene, Shizuoka (H. Nagaoka)

**Keywords:** ehrlichiosis, bacteria, parasites, vector-borne infections, zoonoses, *Ehrlichia* sp., antibody, seroconversion, P28 outer membrane proteins, Japan

## Abstract

In retrospective analyses, we report 3 febrile patients in Japan who had seroconversion to antibodies against *Ehrlichia chaffeensis* antigens detected by using an immunofluorescence and Western blot. Our results provide evidence of autochthonous human ehrlichiosis cases and indicate ehrlichiosis should be considered a potential cause of febrile illness in Japan.

Human ehrlichiosis is a tickborne infectious disease caused by *Ehrlichia* sp. that has primarily been detected in the United States. Common clinical manifestations of human ehrlichiosis are fever, headache, myalgia, and malaise. Leukopenia and thrombocytopenia often occur. Symptoms range from mild fever to severe illness with multiple organ dysfunction, which is occasionally fatal (*1*). In a retrospective analysis, we show serologic evidence for human ehrlichiosis in 3 febrile patients in Japan.

In case 1, a male patient, who was 48 years of age and worked in the manufacturing industry, sought care at a primary care clinic in 2015 for high fever (>40°C) and headache ≈1 month after hiking in the mountains. The clinic physician prescribed levofloxacin and acetaminophen, but the treatment was not effective. Therefore, the patient was seen at the Japanese Red Cross Wakayama Medical Center. The day before onset of high fever, the patient found a small rash on the left side of his abdomen. This date was considered day 0, although there might have been symptoms that the patient was unaware of before that time. The rash was an erythema migrans–like lesion that expanded on day 5. The patient was hospitalized, and borreliosis or tick-associated rash illness, which is similar to Lyme borreliosis–like erythema migrans, was suspected (*2*); however, a tick bite or eschar was not observed. After intravenous administration of minocycline (200 mg/d), the patient’s fever abated, but the lesion expanded and was accompanied by puritis. On day 10, the patient was discharged from the hospital, after which the rash gradually disappeared. Diagnostic tests for borreliosis were negative. We retrospectively performed immunofluorescence assays (IFAs) and Western blot (Appendix) using patient serum samples collected on days 2 and 17. We showed seroconversion to antibodies against *Ehrlichia chaffeensis* antigens by IFA and the presence of IgM and IgG against *Ehrlichia* sp. P28 protein by Western blot (Table; Figure). We suspected the patient had ehrlichiosis and tick-associated rash illness.

**Table:** Evaluation of immunofluorescence assay titers and Western blots of serum samples from 3 febrile patients demonstrating serologic evidence of human exposure to ehrlichiosis agents in Japan*

Case no. (year)	No. days†	*Ehrlichia chaffeensis* antigens, IgM/IgG				*Anaplasma phagocytophilum* antigens, IgM/IgG		
		IFA, THP-1 cells	Western blot			IFA		Western blot, THP-1 cells
			DH82 cells	THP-1 cells		THP-1 cells	HL60 cells	
1 (2015)	2	20/160	−/+	−/−		<20/<20	<20/<20	−/−
	17	80/640	+/+	+/+		<20/<20	<20/<20	−/−
2 (2018)	14	20/20	+/+	+/+		<20/<20	<20/<20	−/−
	32	40/320	+/+	+/+		<20/<20	<20/<20	−/−
	60	20/20	+/+	+/+		<20/<20	<20/<20	−/−
3 (2018)	5	20/20	+/+	+/+		<20/40	<20/20	−/+
	58	80/80	+/+	+/+		<20/40	<20/40	−/+
	115	20/320	+/+	+/+		<20/40	<20/40	−/+

*Serum samples were collected from 3 patients in Japan in 2015 and 2018 and assayed by using THP-1, DH82, or HL60 cells infected with *E. chaffeensis* or *A. phagocytophilum*. Western blots were categorized as positive or negative for IgM and IgG against antigens from each bacterial species. IFA, immunofluorescence assay. †No. days after onset of illness.

**Figure:**
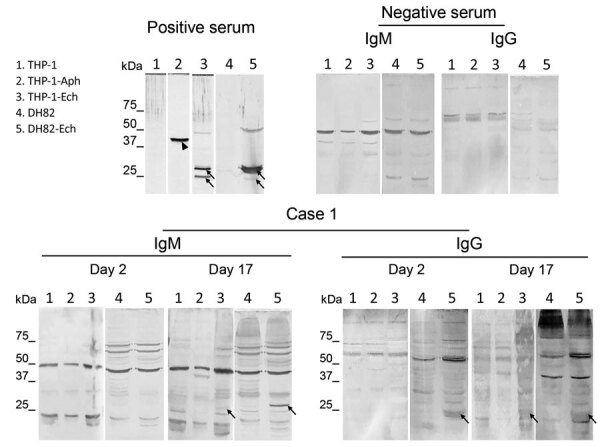
Western blots using serum samples from a febrile patient (case 1) in Wakayama Prefecture in study showing serologic evidence of human exposure to ehrlichiosis agents in Japan. Serum samples were collected from the patient on day 2 and 17 after onset of illness. Human THP-1 and canine DH82 cells were uninfected or infected with *Ehrlichia chaffeensis* . THP-1 cells were also infected with *Anaplasma phagocytophilum*. Cell lysates were separated and Western blot was performed as described (Appendix). We used uninfected THP-1 and DH82 cells as negative lysate controls. We used rabbit serum against recombinant P44 antigens specific for *A. phagocytophilum* and recombinant P28 antigens specific for *E. chaffeensis* (1:10,000 dilution) as positive serum controls. We used serum from a healthy donor as a negative control serum (Precision for Medicine, https://www.precisionbiospecimens.com). The patient’s serum samples and negative control serum were diluted 1:250 and used to probe the blots. We used alkaline-phosphatase-conjugated goat anti-human IgM μ-chain and anti-human IgG γ-chain (Thermo Fisher Scientific, https://www.thermofisher.com) as secondary antibodies. Arrows indicate *E. chaffeensis*-specific P28 antigens (encoded by a *p28* multigene family). Arrowhead shows *A. phagocytophilum*-specific P44 antigen (encoded by a *p44* multigene family).

In case 2, a male patient, who was 66 years of age and worked as a truck driver, sought care at the Ise Red Cross Hospital in 2018 for fever (38°C), annular erythema, and malaise. The patient had renal impairment and jaundice. The principal physician suspected leptospirosis, but diagnostic tests for leptospirosis were negative. The physician suspected other bacterial infections, including Japanese spotted fever (JSF) or anaplasmosis. The patient was treated intravenously with minocycline (200 mg/d) and sulbactam/ampicillin (6 g/d) for 4 days. Subsequently, amoxicillin (1.5 g/d) was administered orally for 14 days, and the patient recovered. Diagnostic tests for JSF were negative. We retrospectively analyzed patient serum samples collected on days 14, 32, and 60 after onset of illness. We showed seroconversion to antibodies against *E. chaffeensis* antigens by IFA and the presence of IgM and IgG against *Ehrlichia* sp. P28 protein by Western blot (Table; Appendix Figure 1). The IFA titers for both IgM and IgG decreased on day 60.

In case 3, a female patient, who was 69 years of age and owned a Japanese-style accommodation, sought care at the Ise Red Cross Hospital in 2018 for mild fever, generalized edema and rash, headache, and malaise. The principal physician suspected JSF and treated the patient with oral minocycline (200 mg/d) and levofloxacin (500 mg/d) for 10 days; the patient recovered. Diagnostic tests for JSF were negative. We retrospectively analyzed patient serum samples collected on days 5, 58, and 115 by IFA and Western blot and found seroconversion to antibodies against *E. chaffeensis* antigens by IFA and the presence of both IgM and IgG against *Ehrlichia* sp. P28 protein antigens by Western blot (Table; Appendix Figure 2). In this case, the IgM titer increased in the convalescent-phase serum on day 58 but decreased on day 115. However, the IgG titer increased on days 58 and 115 after onset of illness. In addition, we detected antibodies against *Anaplasma phagocytophilum* by IFA and *A. phagocytophilum*–specific P44 surface antigen by Western blot. We detected only IgG antibodies against *A. phagocytophilum* in all 3 serum samples, suggesting a past infection with *A. phagocytophilum*.

The 3 patients lived on the Kii peninsula of Japan (Appendix Figure 3), which is known to be a JSF-endemic area, especially in Wakayama and Mie Prefectures (*3*,*4*). In addition, anaplasmosis exists in those areas (*5*). Previously, we revealed the presence of ticks infected with *A*. *phagocytophilum* and *Ehrlichia* sp. that could potentially infect humans in Mie prefecture (*6*,*7*). In particular, members of the *Ehrlichia* sp. genotype 2 group, including *Ehrlichia* sp. MieHl92 and MieHl94, were considered candidate organisms that might cause human ehrlichiosis in Japan (*6*). 

In conclusion, we provide serologic evidence of autochthonous cases of human ehrlichiosis in Japan. We recommend that ehrlichiosis should be considered as a clinical cause of febrile illness in this country.

Appendix Additional information for serologic evidence of human exposure to ehrlichiosis agents in Japan.
