# Supplementary material for: Serologic Evidence of Human Exposure to Ehrlichiosis Agents in Japan
Source: Emerg Infect Dis. 2022 Nov;28(11):2355–7. doi: 10.3201/eid2811.212566 (PMC9622265; doi:10.3201/eid2811.212566)
Supplement: Appendix — Additional information for serologic evidence of human exposure to ehrlichiosis agents in Japan. [file 21-2566-Techapp-s1.pdf]

Article DOI: https://doi.org/10.3201/eid2811.212566

Serologic Evidence of Human Exposure to Ehrlichiosis Agents in Japan

Appendix

Materials and Methods

We obtained serum samples from 34 patients with fever of unknown origin from 14 prefectures in Japan during 2008–2021. The serum samples were from acute and convalescent phases. The study was approved by the Ethics Committee of the University of Shizuoka (approval no. 1-27) and Japan National Institute of Infectious Diseases (approval no. 999). The samples were retrospectively analyzed for human ehrlichiosis and anaplasmosis by immunofluorescence assay (IFA) and Western blotting by using *Ehrlichia chaffeensis*-infected THP-1 cells, *E. chaffeensis*-infected DH82 cells, *Anaplasma phagocytophilum*-infected THP-1 cells, and *A. phagocytophilum*-infected HL60 cells as antigens. The procedures for IFA and Western blotting were described previously (*1*,*2*).

Results of Laboratory Tests for 3 Patients

Laboratory test results (reference values) for patient 1 (case 1) on day 2 after onset of high fever were: leukocytes, 7.8×10^3 cells/µL ($3.8–9.0 \times 10^3$ cells/µL); thrombocytes, 18.2×10^4 cells/µL ($15.0–40.0 \times 10^4$ cells/µL); lactate dehydrogenase, 281 U/L (124–222 U/L); and C-reactive protein, 5.46 mg/dL (<0.10 mg/dL). Test results (reference values) for patient 2 (case 2) on day 14 after onset of illness were: leukocytes, 9.4×10^3 cells/µL ($3.8–9.0 \times 10^3$ cells/µL);

thrombocytes, 18.5 × 10^4 cells/µL (15.0–40.0 × 10^4 cells/µL); aspartate aminotransferase, 147 U/L (8.0–38.0 U/L); alanine aminotransferase, 239 U/L (4.0–44.0 U/L); C-reactive protein, 11.56 mg/dL (<0.10 mg/dL); bilirubin, 4.3 mg/dL (0.2–1.2 mg/dL); creatinine, 1.5 mg/dL (0.60–1.10 mg/dL). Test results (reference values) for patient 3 (case 3) on day 5 after onset of illness were: leukocytes, 4.5 × 10^3 cells/µL (3.8–9.0 x 10^3 cells/µL); thrombocytes, 12.8 × 10^4 cells/µL (15.0–40.0 × 10^4 cells/µL); aspartate aminotransferase, 40 U/L (8.0–38.0 U/L); alanine aminotransferase, 25 U/L (4.0–44.0 U/L); lactate dehydrogenase, 451 U/L (124–222 U/L); and C-reactive protein, 0.69 mg/dL (<0.10 mg/dL).

Discussion

In Asia, cases of human ehrlichiosis are extremely rare. Serologic evidence of human ehrlichiosis was reported in South Korea (2 cases), and serologic evidence and PCR detection of ehrlichiosis was reported in Taiwan (2 cases) (*3*–*5*). Epidemiologic surveillance has shown that antibodies against *Ehrlichia* antigens have been detected in healthy volunteers in Thailand, Japan, and China (*6*–*8*). Thus, human ehrlichiosis is potentially present in Asia, including Japan.

References

1. Ohashi N, Gaowa, Wuritu, Kawamori F, Wu D, Yoshikawa Y, et al. Human granulocytic anaplasmosis, Japan. Emerg Infect Dis. 2013;19:289–92. PubMed https://doi.org/10.3201/eid1902.120855

2. Gaowa, Yoshikawa Y, Ohashi N, Wu D, Kawamori F, Ikegaya A, et al. *Anaplasma phagocytophilum* antibodies in humans, Japan, 2010–2011. Emerg Infect Dis. 2014;20:508–9. PubMed https://doi.org/10.3201/eid2003.131337

3. Heo EJ, Park JH, Koo JR, Park MS, Park MY, Dumler JS, et al. Serologic and molecular detection of *Ehrlichia chaffeensis* and *Anaplasma phagocytophila* (human granulocytic ehrlichiosis agent) in Korean patients. J Clin Microbiol. 2002;40:3082–5. PubMed https://doi.org/10.1128/JCM.40.8.3082-3085.2002

4. Peng SH, Yang SL, Ho YN, Chen HF, Shu PY. Human case of *Ehrlichia chaffeensis* infection, Taiwan. Emerg Infect Dis. 2019;25:2141–3. PubMed https://doi.org/10.3201/eid2511.190665

5. Yen TY, Tung YJ, Wang HC, Tsai KH. Detection of *Ehrlichia chaffeensis* in a febrile patient in Kinmen, an offshore island of Taiwan. J Formos Med Assoc. 2020;119:1329–30. PubMed https://doi.org/10.1016/j.jfma.2019.11.019

6. Heppner DG, Wongsrichanalai C, Walsh DS, McDaniel P, Eamsila C, Hanson B, et al. Human ehrlichiosis in Thailand. Lancet. 1997;350:785–6. PubMed https://doi.org/10.1016/S0140-6736(05)62571-8

7. Kawahara M, Ito T, Suto C, Shibata S, Rikihisa Y, Hata K, et al. Comparison of *Ehrlichia muris* strains isolated from wild mice and ticks and serologic survey of humans and animals with *E. muris* as antigen. J Clin Microbiol. 1999;37:1123–9. PubMed https://doi.org/10.1128/JCM.37.4.1123-1129.1999

8. Zhang L, Shan A, Mathew B, Yin J, Fu X, Zhang J, et al. Rickettsial seroepidemiology among farm workers, Tianjin, People's Republic of China. Emerg Infect Dis. 2008;14:938–40. PubMed https://doi.org/10.3201/eid1406.071502

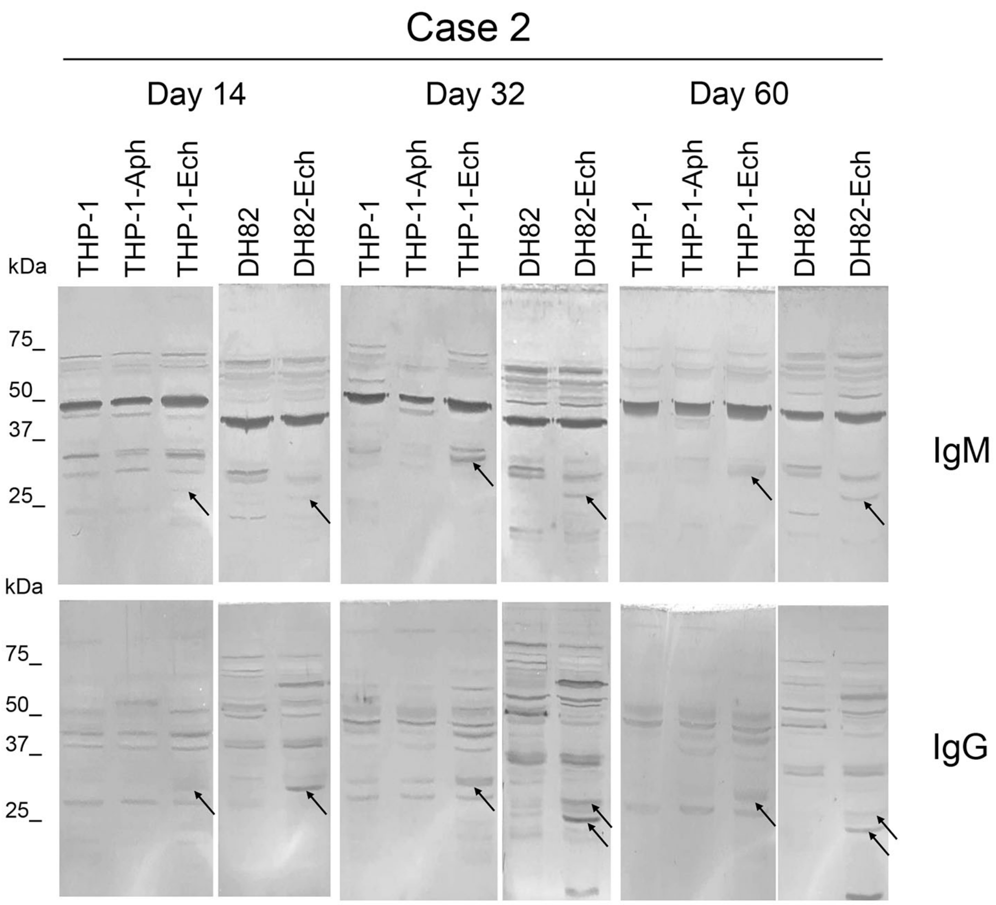

Appendix Figure 1. Western blots using acute-and convalescent-phase serum samples from a febrile patient (case 2) in Mie prefecture in study showing serologic evidence of human exposure to ehrlichiosis agents in Japan. Serum samples were collected from the patient on days 14, 32, and 60 after onset of illness. Human THP-1 and canine DH82 cells were uninfected or infected with *Ehrlichia chaffeensis* (Ech). THP-1 cells were also infected with *Anaplasma phagocytophilum* (Aph). We used uninfected THP-1 and DH82 cells as negative lysate controls. The patient’s serum samples were diluted 1:250 and used to probe the blots. We used alkaline-phosphatase-conjugated goat anti-human IgM μ-chain and anti-human IgG γ-chain antibodies (Thermo Fisher Scientific, https://www.thermofisher.com) as secondary antibodies. Arrows indicate *E. chaffeensis*-specific P28 antigens (encoded by a *p28* multigene family).

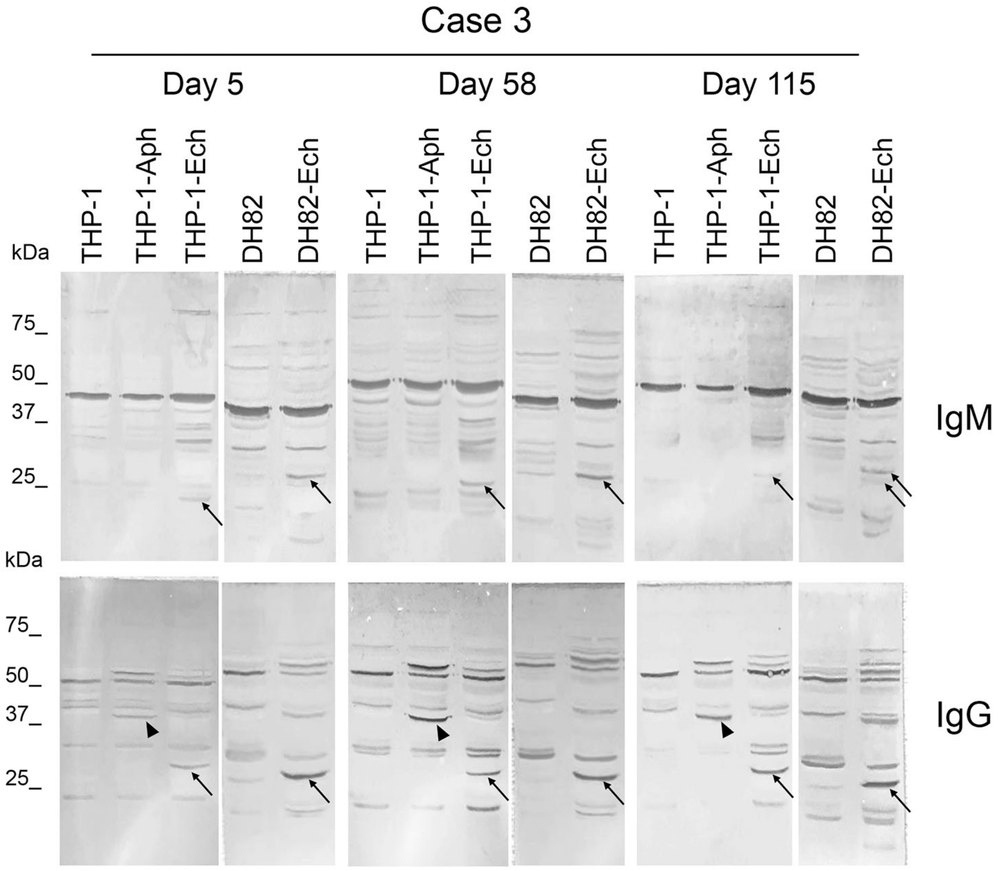

Appendix Figure 2. Western blots using acute-and convalescent-phase serum samples from a febrile patient (case 3) in Mie prefecture in study showing serologic evidence of human exposure to ehrlichiosis agents in Japan. Serum samples were collected from the patient on days 5, 58, and 115 after onset of illness. Human THP-1 and canine DH82 cells were uninfected or infected with *Ehrlichia chaffeensis* (Ech). THP-1 cells were also infected with *Anaplasma phagocytophilum* (Aph). We used uninfected THP-1 and DH82 cells as negative lysate controls. The patient’s serum samples were diluted 1:250 and used to probe the blots. We used alkaline-phosphatase-conjugated goat anti-human IgM μ-chain and anti-human IgG γ-chain antibodies (Thermo Fisher Scientific, https://www.thermofisher.com) as secondary antibodies. Arrows indicate *E. chaffeensis*-specific P28 antigens (encoded by a *p28* multigene family). Arrowheads show *A. phagocytophilum*-specific P44 antigens (encoded by a *p44* multigene family).

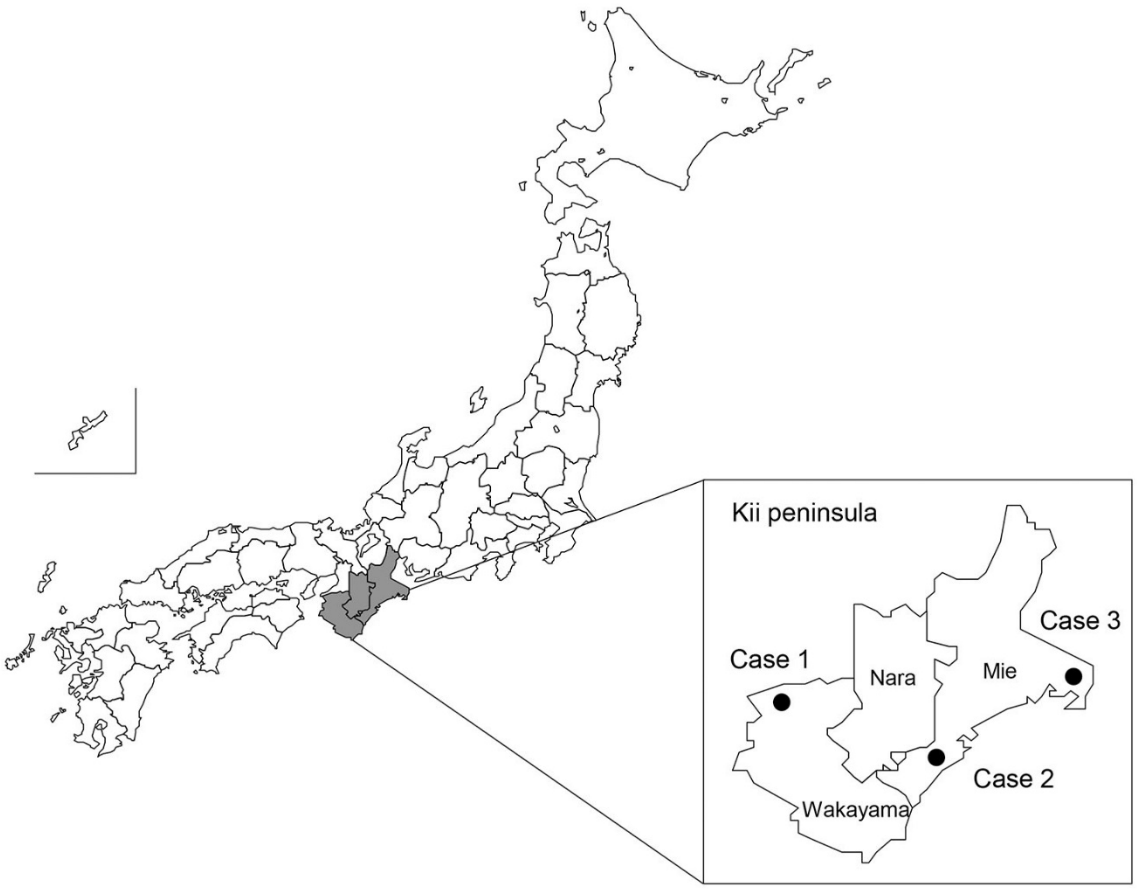

Appendix Figure 3. Map of Japan showing residential locations of 3 febrile patients who had seroconversion to antibody against E. chaffeensis antigens in study of serologic evidence of human exposure to ehrlichiosis agents. Serum samples were collected from these patients in 2015 and 2018. Kii peninsula is known to be highly endemic for Japanese spotted fever, especially Wakayama and Mie prefectures, and anaplasmosis is also present. Closed circles indicate where each patient lived at the time of serum collection.
